# Diffuse fasciitis with eosinophilia in boys: a case-based review

**DOI:** 10.1007/s00296-023-05362-x

**Published:** 2023-06-14

**Authors:** Agnieszka Wosiak, Małgorzata Biernacka-Zielińska, Justyna Roszkiewicz, Elżbieta Smolewska

**Affiliations:** Department of Paediatric Cardiology and Rheumatology, Central Clinical Hospital of the Medical University of Lodz, 36/50 Sporna St., 91-738 Lodz, Poland

**Keywords:** Diffuse fasciitis with eosinophilia, Eosinophilic fasciitis in children, Hypercalcemia, Clinical presentation of eosinophilic fasciitis

## Abstract

Diffuse fasciitis with eosinophilia (EF) is a rare condition classified as a part of the connective tissue disorders. The clinical presentation of this condition can be diverse, however the main symptoms include symmetrical swelling and hardening of distal parts of limbs accompanied by peripheral eosinophilia. The diagnostic criteria are not specified. In inconclusions cases Magnetic Resonance Imaging (MRI) and skin to muscle biopsy may be useful. The pathogenesis and ethiology remain unknown, but extensive physical exertion, certain infectious factors, such as *Borrelia burgdorferi*, or medications may serve as a trigger. EF affects equally women and men, mainly in their middle age, however the disease can occur at any age. The standard therapy contents gluccocorticosteroids. As a second-line treatment, methotrexate is usually chosen. In this article we compare world reports of EF in paediatric patients with the cases of two adolescent male patients recently hospitalized in the Department of Paediatric Rheumatology.

## Introduction

Diffuse fasciitis with eosinophilia (EF) is an uncommon scleroderma-like disease which was described for the first time in 1974 by Shulman [1]. Until recently it was believed that there is a higher prevalence of the disease in male sex. However, latest reviews prove that the disease affects women as often as men. The peak of morbidity falls on the age 47–57 years [2]. Cases found in paediatric patients are rare, but have been documented (Table 2). So far, the disease etiology is unknown, but a number of triggers have been reported, such as intense exercise, certain medications, infectious agents, autoimmune diseases, physical factors and hematologic disorders. The pathogenesis has also not been fully understood. An abnormal immune response leading to excessive release of eosinophils seems to be the main cause of symptoms. Subsequently, degranulation of eosinophils generates the consequent fibrosis [3]. Additionally, the interaction between eosinophils and fibroblasts leads to production of fibrogenic cytokines such as transforming growth factor-β (TGF-β), tumor necrosis factor alpha (TNF-α), interleukin-1 (IL-1) and interleukin-6 (IL-6) [30, 31]. The consequences of these processes are tissue changes presenting as swelling and painful erythema of the distal parts of the limbs progressing to their induration and stiffness. As a result, myalgia and proximal muscle weakness may appear. The characteristic features are “peaud’orange” appearance of the skin or protrusion of the veins and vascular pattern known as “groove sign”[32]. The patient may report general malaise or weight loss [31]. Internal organ involvement is rare, but abnormalities of the pericardium, pulmonary tissue or kidneys have been also reported [3]. The diagnostic criteria include: thickening of the fascia found in magnetic resonance imaging (MRI), fibrosis of the subcutaneous connective tissue, thickening of the fascia and cellular infiltration of eosinophils and monocytes in skin-to-muscle biopsy (including fascia) [31, 33, 34]. Authors emphasize that Raynaud’s phenomenon as well as nailfold capillary abnormalities are not typical for EF [2, 30, 32, 35].

Typical abnormalities found in basic laboratory tests include eosinophilia in peripheral blood, elevated erythrocyte sedimentation rate (ESR) and C-reactive protein (CRP) along with polyclonal hypergammaglobulinemia [3, 31, 33, 36]. Hypercalcemia may appear in the course of EF but its prevalence is relatively low. The relationship between those two manifestations is unknown. One of the theories states that cytokine release causes both inflammatory cell activation resulting in inflammation and eosinophilia, and osteoclastic bone resorption leading to hypercalcemia [15, 25].

So far there is no standard treatment protocol for EF. Moreover, some patients do improve spontaneously without any medications. In more severe cases, systemic glucocorticosteroids (GCS) treatment is advised (0.5–1.0 mg/kg/day for prednisone). Second line therapy includes low doses of methotrexate (MTX). Alternatively, dapsone, tacrolimus or cyclosporine may be applied. In the medical literature, there are reports of successful use of TNF-α inhibitors, such as adalimumab and etanercept or IL-6 inhibitors like tocilizumab [37] in the treatment of refractory EF. Moreover, the importance of treating the triggering factor is often emphasised. The use of antihistamine drugs [4] and superficial ointments, such as topical tacrolimus may be also helpful. Pharmacological treatment should be supported by intensive physiotherapy [2, 31–33].

## Methods

The objective of this article was to present different aspects of diffuse fasciitis in children. We performed extensive literature review of PubMed and Scopus databases using “diffuse fasciitis with eosinophilia”, “eosinophilic fasciitis”, “clinical presentation of eosinophilic fasciitis” and “eosinophilic fasciitis in children” as keywords. Only articles written in English and published in last 20 years were included in the further analysis. The literature review inclusion criteria were diffuse fasciitis diagnosis in children under 18 years old on the basis of clinical manifestation, laboratory tests, biopsy or MRI [4–29]. The articles which described patients over 18 years old were used only to present the latest knowledge about diffuse fasciitis, however, were excluded from literature review [1–3, 30–39].

### Case I

**Fig. 1:**
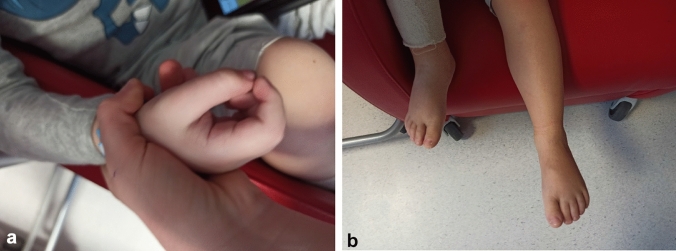
**a** Contracture of the right hand, **b** swelling of right foot

A 4-year-old boy presented with fever, aggravation of his general condition, oedema of abdomen, hands and feet associated with hardening of skin of distal parts of legs and erythema of the facial skin was admitted to the Department of Paediatric Rheumatology (Fig. 1). Due to progression of symptoms, the boy refused to walk. His medical history included autism.

**Fig. 2:**
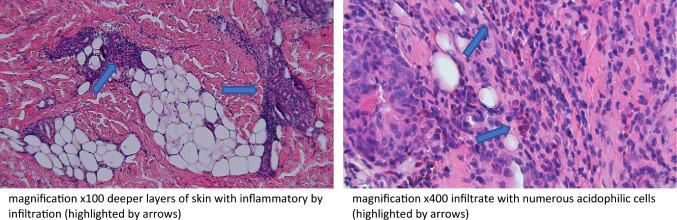
Histopathological examination of skin-muscle biopsy (Jozef Kobos, MD, PhD. Department of Pathomorphology, Medical University of Lodz)

Laboratory tests performed on admission revealed elevated leucocytosis with eosinophilia reaching 48.1%, thrombocythemia, slightly elevated CRP, lactate dehydrogenase activity (LDH), D-Dimers and immunoglobulin E. Vitamin D level was significantly decreased. The antinuclear antibodies (ANA) and antineutrophil cytoplasmic antibodies (ANCA) were at the positive limit (Table 1). The ultrasound of abdomen, lower and upper limbs as well as echocardiography showed no abnormalities. The pulmonary high-resolution computed tomography (HRCT) was performed, with no evidence of pathology. Proliferative diseases were excluded on the basis of bone marrow biopsy. The patient was treated with empirical antibiotic (cefuroxime) and anti-parasitic treatment with no clinical improvement. The suspicion of EF was raised. Histopathological biopsy of skin and fascia from patient’s lower limb was performed, confirming the diagnosis of EF (Fig. 2).

**Table 1:** Laboratory tests results of patient 1 and 2 on admission to the hospital

	Patient 1	Patient 2	Reference normal range
WBC [10^3^/μl]	17.6	15.77	4–12
Haemoglobin [g/dl]	10.8	10.2	12–15.5
Haematocrit [%]	32.2	29.2	37–44
Platelets [10^3^/μl]	470	368	150–400
Eosinophilia [10^3/^μl]	8.47	2.32	0.1–0.5
Eosinophilia [%]	48.1	20.0	2–4
CRP [mg/l]	11.70	1.6	< 0.5
ESR [mm/h]	40	51	0–10
D‑dimer [ng/ml]	5131.88	1050.00	0–500
LDH [U/l]	436	219	170–283
Immunoglobulin IgE [KU/l]	182	147	< 56
Immunoglobulin IgA [ng/ml]	0.747	163	0.63–3.32
Immunoglobulin IgM [g/l]	0,70	18,50	0.4–1.98
Immunoglobulin IgG [g/l]	7.661	1.36	6.38–17.00
Vitamin D [ng/ml]	17.7	9.4	30–100
Calcium [mg/dl]	9.30	14.12	9.16–10.52
Parathormon [pmol/l]	No data	1.09	2.32–9.28
ANA	1:80 fine-grained spotted	1:640 spotted	< 1:80
ANCA	1:20 pANCA atypical	Negative	< 1:20

*WBC* white blood cells, *CRP* C-reactive protein, *ESR* erythrocyte sedimentation rate, *LDH* lactate dehydrogenase, *ANA* antinuclear antibodies, *ANCA* antineutrophil cytoplasmic antibodies

The patient was treated with systemic GCS starting from intravenous methylprednisolone, followed by oral prednisone. MTX was applied as the disease-modifying treatment. Course of intensive physiotherapy was also applied. After 10 months of therapy, due to lack of significant improvement and recurrent rash, methotrexate was changed to mycophenolate mofetil (MMF). The initial improvement was interrupted by systemic steroid dose reduction and recurrent, severe respiratory tract infections. Due to the deterioration of patient’s general condition, subfebrile state and muscle pain treatment with intravenous immunoglobulins (IVIG) at a dose of 1 g/kg once a month was introduced. Early follow-up is very promising. The remission of erythema of the fascial skin, oedema of hands and feet, as well as improvement of motor skills were obtained. The normalization of laboratory tests is likewise observed.

### Case II

An 11-year-old boy without significant medical history was admitted to the Department of Paediatric Rheumatology due to headache lasting for 4 months, deterioration of the general condition, apathy, polyuria, constipation, oedema of feet and hands, and decrease of muscle strength.

Laboratory tests revealed mild anaemia, iron deficiency, eosinophilia, accelerated ESR, low vitamin D level, hypergammaglobulinmia, hypercalcemia, hypophosphatemia, elevated urea and creatinine concentration. A high calcium creatinine index and reduced parathormone levels were observed (Table 1).

**Fig. 3:**
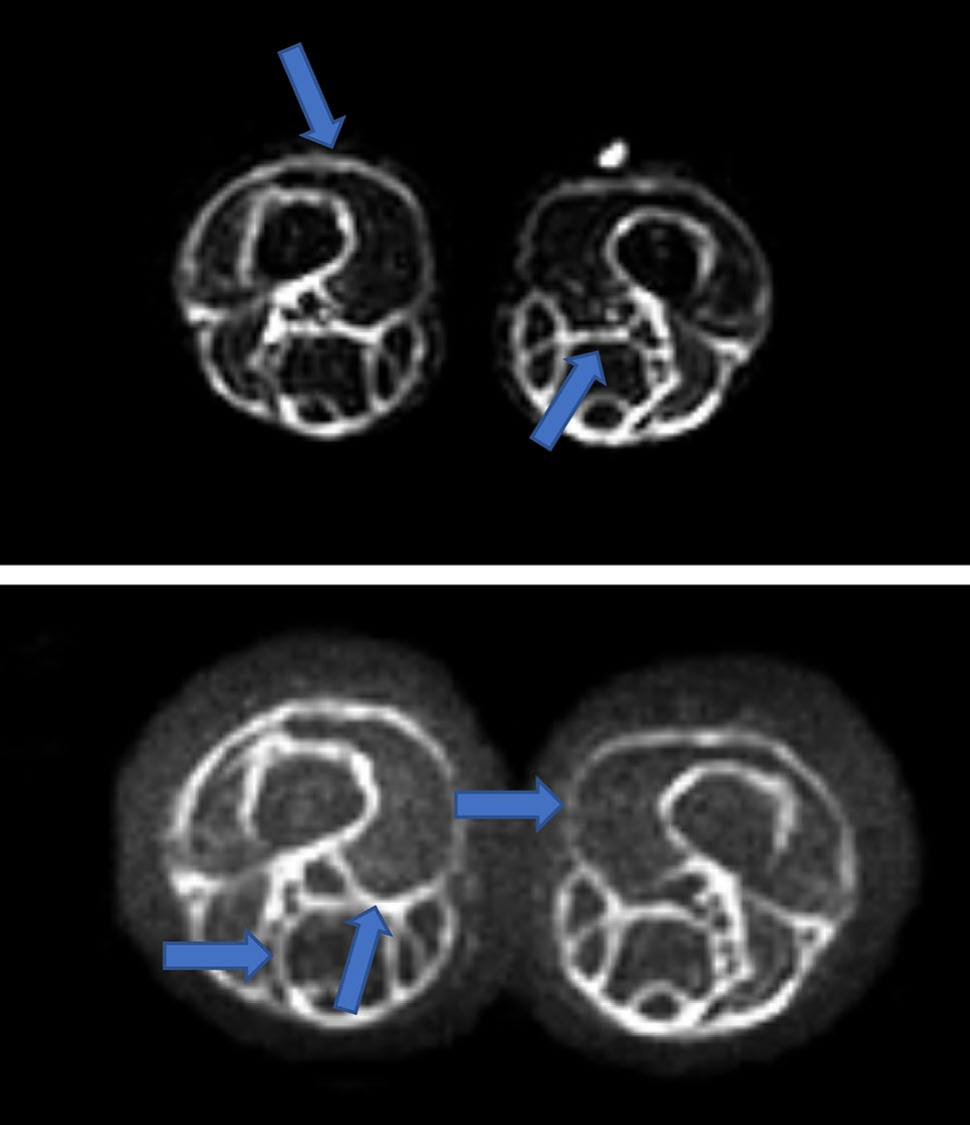
Hyperintense signal of fascia of lower limbs showed in MRI (highlighted by arrows)

**Fig. 4:**
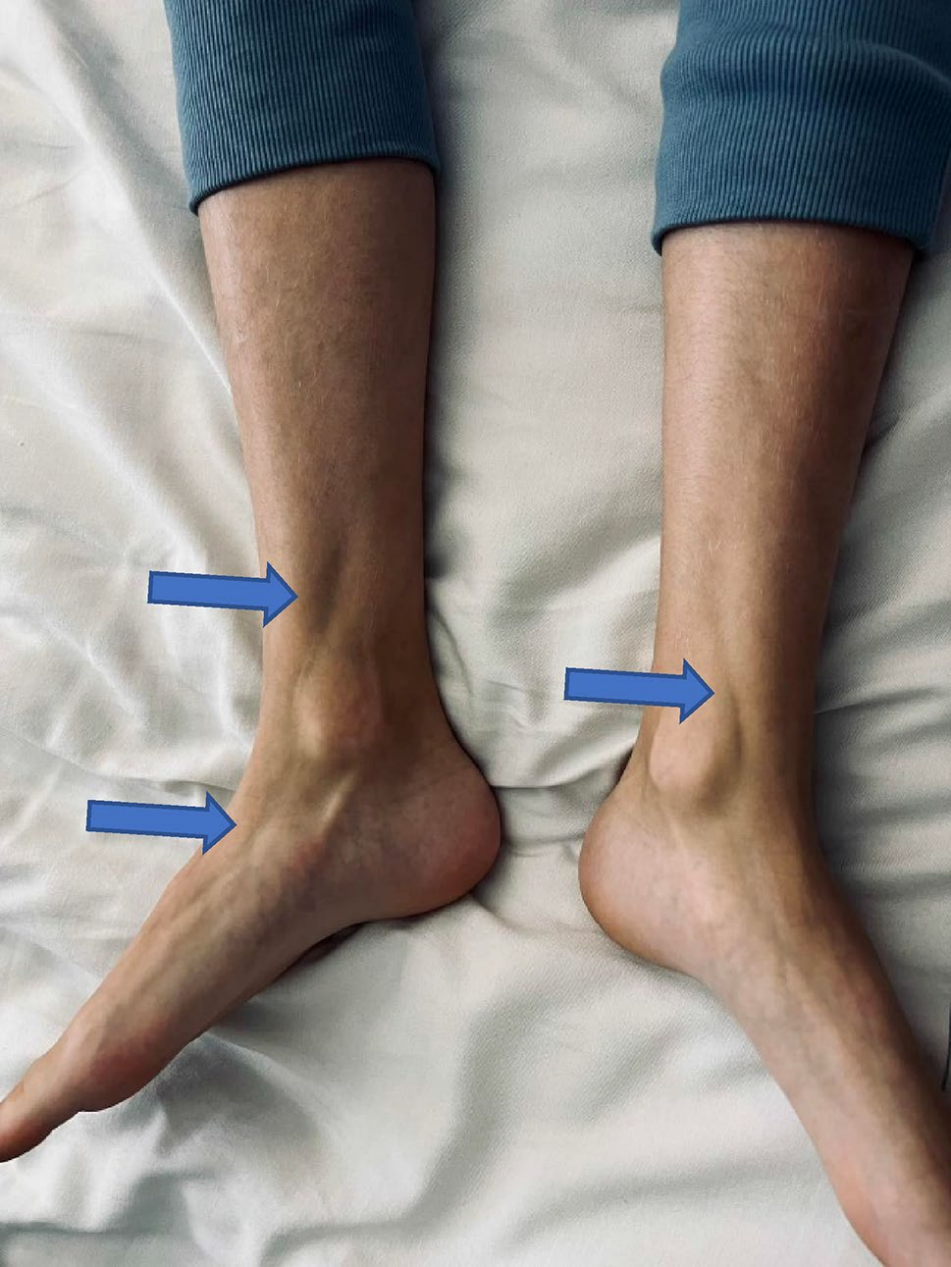
Muscle atrophy with typical groove sign (highlighted by arrows)

The MRI of the head did not reveal any abnormalities. Abdominal ultrasound showed thickening of the renal parenchymal layer and enlargement of the kidneys. Thyroid and parathyroid ultrasound showed no abnormalities. The patient was treated with furosemide and infusion fluids without any improvement. In addition, a subfebrile state was observed. The patient underwent further examination. On subsequent abdomen ultrasound enlargement of kidneys with hyperechogenic signal of renal parenchymal and enlargement of the liver persisted. A whole-body MRI revealed hepatomegaly and hyperintense signal of kidneys and lower limbs fascia (Fig. 3). Bone scintigraphy and nailfolds capillaroscopy showed no pathology. Proliferative diseases were excluded on the basis of bone marrow biopsy. Finally, a fascia and muscle biopsy was performed, confirming the diagnosis of diffuse fasciitis with eosinophilia. The patient was treated with furosemide and infusion fluids, bisphosphonates, angiotensin-converting enzyme inhibitor, macrogols, intravenous and oral GCS and MTX. Course of intensive physiotherapy was also applied. After the first line of therapy, the patients’ general condition improved and the normalisation of laboratory parameters’ values was observed. However, during follow-up visits sclerosis and contraction of the limbs with muscle weakness and groove sign were still observed (Fig. 4). For this reason it was decided to start MMF therapy, with the need for further close observation of the patient. Despite intensive treatment, headache has not resolved. Due to the possible effect of the medication such as captopril on symptoms, it was decided to exclude it from the treatment. The patient was referred to neurologist to continue the diagnostic process of the headache.

## Discussion

EF is a rare disease entity in the paediatric population. Its exact prevalence is unknown and only a few dozen cases of this phenomenon in children have been described (Table 2). The disease may be triggered by several factors. Intensive exercise was documented in three cases [10, 12, 15], infectious agents (*Borrelia burgdorferi, Mycoplasma pneumoniae, Parvovirus B19)* in four cases [7–9, 28], followed by other autoimmune diseases in one case [15] and hematologic disorders in one case [23]. There was one patient with medical history of emotional tension [18], one case with confirmed exposure to a chemical and biological agents [21] and in one case the symptoms were preceded with surgery [29] (Table 2). Other triggers such as certain drugs (statins, phenytoin, ramipril, heparin) or haemodialysis treatment are mentioned, however, did not appear in collected literature review. Three patients had positive family history of EF or other rheumatic disease [6, 24, 26]. None of our patients had a documented cause of the disease onset. The first patient had a history of autism and the second one was physically active, however, he did not confirm intensive training before the appearance of symptoms. The characteristic swelling of the limbs, thickening of the muscles and fascia with flexion contractures were not mentioned only in two cases [12, 29] (Table 2). A systemic involvement features, such as general weakness, fever or subfebrile state are symptoms characteristic for childhood-onset EF and are documented in seven cases [7, 9, 15, 19, 21, 26, 28]. Other symptoms including reddening of the face (patient 1) or groove sign (patient 2) are also common findings [3, 38] (Table 2). The headache, polyuria and constipation reported by the second patient should rather be related to hypercalcaemia. The abnormalities in laboratory tests mostly include eosinophilia in peripheral blood and it was confirmed in almost 69% of patients, elevated ESR and hypergammaglobulinemia was mentioned in 34% (Table 2). Symptomatic hypercalcemia not responding to the initial treatment regimen was a major clinical challenge in the case of patient no. 2. To date, there are two other reports of EF and hypercalcemia in children [15, 25]. Both occurred in young boys, one of whom had a history of hypothyroidism. Vitamin D and parathyroid hormone levels were not elevated, and there were no visible changes on bone imaging studies, what suggests an idiopathic occurrence of hypercalcemia in each case (Table 2).

**Table 2:** Literature review of paediatric patients diagnosed with diffuse fasciitis with eosinophilia

Authors	Main complaints	Medical history	Confirmation in Biopsy	Confirmation in USG	Confirmation in MRI	Laboratory tests abnormality	Medical Treatment
A. Uckun et al. [4]	Swelling, tenderness, erythema and induration of arm, joint mobility constraint	No past history	+	No data	No data	Eosinophilia	Oral Hydroxyzine
A. I. Rodriguez Bandera et al. [5]	Induration of the skin of thigh and buttock, peau d’orange	No past history	No data	+	No data	Mild eosinophilia	GCS, MTX
T. Wu Tiffany et al. [6]	Stiffness of forearms, pain with extension of the fingers, thickening of the skin, contracture of the joints	Positive family history- rheumatoid arthritis in the patient's mother, with possible EF and scleroderma overlap	+	No data	+	Eosinophilia, elevated serum aldolase	GCS (intravenous and later oral prednisolone), MTX, Imatinib
T.M. Sarah et al. [7]	Decreased energy, weight loss, cough, dyspnoea, malaise, decreased appetite, abdominal pain, pale, night sweats, fever, lymphadenopathy, swelling and stiffness of joints	Confirmed Parvovirus B19 infection	+	No data	No data	Anaemia, eosinophilia, thrombocytosis, elevated ESR, CRP, hypoalbuminemia	IGIV, GCS, NSAID (Naproxen), MTX, Etanercept, Adalimumab
M. Antic et al. [8]	Oedema, flaking skin, myalgia, contractures of joints	Mycoplasma-related pneumonia	+	No data	No data	Eosinophilia, hypergammaglobulinemia, positive antinuclear antibodies, increased leukocytes	Prednisone
R. Papa et al. [9]	Motility limitations and contractures of joints, asthenia, low-grade fever and severe stiffness at upper limbs, hepatosplenomegaly with lymphadenopathy, rhinolalia with inability of swallow	Respiratory infection, Mycoplasma-related pneumonia	+	No data	+	Elevated CRP, ESR, eosinophilia, hypergammaglobulinemia, increased triglycerides, anti-GDA antibodies, slightly positive ANA	Prednisone, Methylprednisolone infusion, Cyclosporine, MTX
T. Ergun et al. [10]	Stiffness of both arms and neck	Intensive violin practising	+	No data	+	Elevated CRP, hypergammaglobulinemia, positive antinuclear antibody	GCS, MTX
L. Dziadzio et al. [11]	Unilateral forearm swelling, Stiffness of elbow	No past history	+	No data	+	Eosinophilia, elevated Il-5 and TGF-β	Prednisone
C. Alexanian et al. [12]	Painful plaque on right thigh	Intensive training	+	No data	No data	No data	Minocycline and Clobetasol ointment, Prednisone
C. M. Hedrich et al. [13]	Localised thickening and induration of the skin, polyarthritis	No data	+	No data	+	Eosinophilia, hypergammaglobulinemia, elevated eosinophilic cationic protein and ESR	Prednisone, MTX
P. Huppke et al. [14]	Stiffness of joints	No past history	+	+	+	Eosinophilia, hypergammaglobulinemia	Pulsed Methylprednisolone
U. Namita et al. [15]	Tightening and swelling of both forearms, morning stiffness, weight loss	Hypothyroid Intense training before first symptoms	No data	+	No data	Eosinophilia, hypergammaglobulinemia, Elevated CRP and ESR, hypercalcemia, raised ACE levels, vitamin D deficiency	GCS, MTX
Y. Kan et al. [16]	Skin thickening, joint stiffness, hypopigmented patches with atrophy in the neck and trunk and cutaneous sclerosis	No past history	+	No data	No data	Eosinophilia	Methylprednisolone, MTX
A. B. Lese et al. [17]	Pain, swelling, erythema and cellulitis from left hand to forearm	No past history	+	No data	+	Elevated CRP	Arthrotomy, Clindamycin, Vancomycin and Piperacillin/Tazobactam, NSAID
Y. Liu et al. [18]	Swelling, myalgia, stiffness, tightness and aching in muscles	Emotional tension	+	No data	+	Eosinophilia, hypergammaglobulinemia, elevated CRP, ESR	Systemic GCS
K. Loupasaki et al. [19]	Fatigue, weight loss, tightness of skin, peau d’orange changes, joint contractures, induration of the skin	No past history	+	No data	+	Eosinophilia, elevated platelet count and ESR	Oral Prednisone, Pulsed Methylprednisolone, MMF
M. Verenes et al. [20]	Stiffness and mild aching in the forearms, peau d’orange changes, induration of the skin	No data	+	+	Not performed	Eosinophilia	Prednisolone
A.G. Ortega-Loyaza et al. [21]	Stiffness, thickening of the skin, limitation of mobility of joints, diffuse hair loss, weight loss	Exposure to a chemical and biological agents	+	No data	+	Eosinophilia, anemia, hypergammaglobulinemia, elevated aldolase level, serum urea nitrogen, proteinuria and hematuria	Prednisone, MTX
S. Pillen et al. [22]	Painless limitation of movement with stiffness of fingers, thickening of the skin, reduced muscles force	No data	+	+	+	Eosinophilia, elevated IgG level and slightly decreased IgA level	Prednisolone, MTX
A. Pituch-Noworolska et al. [23]	Pain and cramps of the right lower thigh with erythema and swollen of the skin	X-linked agammaglobulinemia Injury before episode	+	+	+	Elevated CRP	NSAID, GCS, IVIG, MTX, MMF
N. Poliak et al. [24]	Diffuse oedema and erythema of the face, abdomen and extremities, Induration of the skin, peau d’orange changes	EF in first line cousin	No data	+	+	Eosinophilia	Antihistamines, oral GCS, MTX Methylprednisolone pulses, Infliximab
M. M. Rutter et al. [25]	Painless symmetric swelling of wrists and ankles, with restriction of joints polyuria, nocturia	No data	+	No data	+	Eosinophilia, anaemia, hypercalcemia, increased ESR, creatinine, ACE, aldolase and blood urea nitrogen (BUN), elevated urinary calcium excretion	GCS, MTX
C. Sullivan et al. [26]	Stiffness and swelling of joints, general malaise and weight loss, skin induration	Confirmed illness in siblings	+	No data	Performed with normal imaging	Eosinophilia, elevated IgG, ESR, HLA-A2-positive	Prednisolone, MTX
N. Tzaribachev et al. [27]	Mobility limitation with pain, skin thickening, peau d’orange	No medical history	+	No data	Performed with no confirmed arthritis	Eosinophilia, elevated ESR, CRP, positive ANA 1:160, low-grade positive antibodies against nucleoli	Prednisolone, Methylprednisolone, MTX, Infliximab
A. I. Quintero-Del-Rio et al. [28]	Tightening, dryness, pigmentation of the skin, muscles weakness, loss of weight Raynaud ‘s phenomenon, generalised fatigue, peau d’orange	Urinary tract infection	+	No data	No data	Leucocytosis, eosinophilia, anaemia, elevated ESR, LDH, aldolase	Prednisone, MTX
O. Shamriz et al. [29]	Myalgia	Tonsillectomy	+	No data	Not performed	Elevated creatine phosphokinase	No data

*GKS* glucocorticosteroids, *MTX* methotrexate, *IVIG* intravenous immunoglobulins, *NSAID* non-steroidal anti-inflammatory drugs, *MMF* mycophenolate mofetil

Full-thickness muscle biopsy, which was performed in both patients, is effective in the diagnosis of EF. Other imaging tests of the fascia, such as ultrasound or MRI are helpful in the diagnosis. The hyperintense signal of limbs fascia in whole-body MRI in the second case could have resulted from inflammation. As the absence of Raynaud’s phenomenon is helpful in distinguishing EF from scleroderma, nailfold capillaroscopy was performed in the second patient excluding the presence of microcirculation abnormalities [31, 33].

Almost all available studies indicate GCS as a first-line treatment, what was the treatment of choice in both cases [5–16, 18–28]. When the effects are insufficient, MTX is often introduced, especially in the younger patients [5–7, 9, 10, 13, 15, 16, 21–28]. Other frequently chosen drugs include cyclosporine [9], cyclophosphamide [36] and MMF [19, 23]. Improvement after IVIG treatment was also described [7, 23]. Recently, there is an increasing number of reports of TNF alfa blockers successful use in EF [6, 7, 24, 27]. Moreover, physiotherapy is an important part of the treatment regimen [8, 9, 14, 17, 19, 21–23, 28]. There are studies indicating spontaneous resolution of symptoms [3, 8, 19, 27, 29, 36]. Nevertheless, even in those patients the application of systemic GCS is often necessary in the later stage of the disease. Both of our patients presented good treatment response in early follow-up, however, a relapse of symptoms has occurred and the introduction of the second line of treatment was required.

## Conclusion

EF in children is currently a poorly understood disease. A study describing a 63-person cohort noted that it takes about 11 months from the first symptoms of the disease to the final diagnosis of EF [39]. Due to its low prevalence, the criteria of disease diagnosis and treatment guidelines are not established. Therefore, the publication of new clinical cases can be valuable for raising awareness of such a rare disease. Further research should be undertaken to expand the knowledge of this disease entity.
